# Mapping the spatial distribution of harmful umbilical cord stump care among neonates in Ethiopia: A spatial with multilevel analysis

**DOI:** 10.1371/journal.pone.0310471

**Published:** 2024-10-24

**Authors:** Berihun Bantie, Natnael Moges, Worku Awoke, Abebaw Gedef Azene

**Affiliations:** 1 Department of Comprehensive Nursing, College of Health Sciences, Debre Tabor University, Debre Tabor, Ethiopia; 2 Department of Pediatrics and Child Health Nursing, College of Health Sciences, Debre Tabor University, Debre Tabor, Ethiopia; 3 Department of Epidemology and Biostatistics, School of Public Heath, College of Medicine and Health Science, Bahirdar University, Bahirdar, Ethiopia; Wachemo University, ETHIOPIA

## Abstract

**Introduction:**

The umbilical cord (UC) serves as the main pathway for bacteria to reach the neonate’s body, potentially causing local and severe infections, sepsis, and even death. Consequently, neonatal mortality remains a significant public health concern, particularly in Ethiopia. The World Health Organization (WHO) recommends that the umbilical cord stump be kept clean and dry, with the exception of applying topical antiseptics. However, various harmful substances are still applied to the umbilical cord of neonates. Data on the geographical distribution and risk factors for harmful umbilical cord stump (UCS) care are scarce. Therefore, this study aims to fill this gap.

**Methods:**

A secondary data analysis of the Ethiopian Demographic Health Survey (EDHS 2016) was conducted using a weighted sample of 7,168 live births. ArcGIS version 10.7.1 software was utilized to visualize the spatial distribution of harmful umbilical cord stump (UCS) care practices in Ethiopia. Additionally, a Bernoulli probability model-based spatial scan statistic was applied using Kulldorff’s SaTScan version 9.6 software to identify significant clusters of harmful UCS care. A multilevel logistic regression model was used to determine the factors associated with UCS care practices in Ethiopia. Statistical significance was declared at a two-sided P-value of < 0.05.

**Results:**

Overall, the prevalence of harmful UCS care in Ethiopia was 15.09% (95% CI: 13.9–16.3), with significant spatial heterogeneity across geographical areas. The hotspot areas of harmful US care were observed in the eastern (Somali) and northern (Tigray and Amhara) parts of Ethiopia. In spatial scan analysis, the most likely primary clusters were observed in South Nation Nationalities and Peoples region (SNNPR), secondary clusters in the Somali, tertiary clusters in Tigray, and the next clusters in the Amhara regions, respectively. In the final multilevel model, maternal age (Adjusted odds ratio/AOR 1.07, 95% CI: 1.02–1.12), institutional delivery (AOR 0.64, 95% CI: 0.42–0.97), female neonates (AOR 1.31, 95% CI: 1.04–1.61), rural residence (AOR 2.18, 95% CI: 1.05–4.52), living in Tigray region (AOR 3.79, 95% CI: 1.38–9.38), living in Somali region (AOR, 2.95% CI: 1.02–8.52), and living in Harari region (AOR 3.51, 95% CI: 1.28–9.60) were identified as a significant factors of harmful US care practice in Ethiopia.

**Conclusion:**

In Ethiopia, the distribution of harmful UCS care practices is non-random and highly clustered in the SNNPR, Somalia, Tigray, and Amhara regions. Both individual and community-level factors were significantly associated with the practice. Special emphasis needs to be provided for neonates from those hot-spot areas and to address the identified predictors of harmful umbilical cord stump care practices.

## 1. Introduction

The umbilical cord (UC) is a soft, flexible structure that acts as a lifeline for the growing fetus during pregnancy. When a baby is born, the umbilical cord’s function is no longer needed. The umbilical cord is then clamped and severed close to the baby’s body, and the umbilical cord stump will left attached to the baby’s body. The stump then gradually dries, shrinks, and falls from the body, usually within 5 to 15 days [1].

After a newborn’s UC is cut, the recommended practice is to put nothing on the stump of the cord and let it dry and fall by itself (dry cord care). In this regard, the World Health Organization (WHO) advocated that the stump should be kept clean and dry except for the application of topical antiseptics like chlorhexidine in areas where home deliveries are very common and the neonatal mortality rate exceeds 30 deaths per 1000 live birth [2,3]. However, due to various religious, cultural, and local perspectives, harmful substances were commonly applied to newborns’ umbilical stumps, particularly in low- and middle-income nations [4,5]. These substances were applied due to unscientific beliefs such as for the purpose of hastening cord healing, facilitating separation of the cord, preventing of pain, infection, or bleeding, or keeping the newborn out of evil spirits or cold air [6–11].

In this regard, harmful umbilical cord care remains a major public health concern due to its association with increased neonatal and infant morbidity and mortality [2,12,13]. Globally, the burden of unhygienic umbilical cord care varies considerably, with the highest incidence observed in low- and middle-income countries (LMICs) [4,13]. For instance, by the end of 2016, harmful substances were applied to the umbilical cord stumps of about 29% of neonates delivered at home in Bangladesh and 46% in Nepal [13]. Likewise, in Africa, unhygienic umbilical cord care practices were common, with over 30% of mothers in Rwanda and 63.7% in Nigeria engaging in such practices [14,15]. Similarly, about 19.9% of newborns in Ethiopia’s four major regions (Amhara, Oromia, SNNPR, and Tigray) had butter and other substances applied to their umbilical cord stumps (UCS) [16]. Moreover, a study conducted at Mizan-Tepi University Teaching Hospital in western Ethiopia revealed that the proportion of unhygienic umbilical cord care was estimated to be 40.8% [17]. In contrast, more than two-thirds of newborns in northern Ethiopia and over 70% of children in eastern Ethiopia experienced unhygienic umbilical cord care practices [18–20].

The application of harmful substances on the umblical cord stump is the main pathway for the bacteria to reach the newborn’s body, causing omphalitis, tetanus, severe sepsis, and finally death [12,13,21,22]. The risk of umbilical cord infection (ompahlitis) is increased by 62% in neonates receiving unhygienic topical umblical-cord care, which can rapidly advance into systemic infections and death [23]. Neonatal infections (sepsis tetanus, meningitis…) in turn were the third leading cause of neonatal death in the world, which accounted for approximately one-third (30%) of all neonatal deaths [24–26]. Nowadays, each year, 2.4 million neonatal deaths are reported in the world [24]. Sub-Saharan Africa (SSA) has the highest neonatal mortality rate in the world, weighing 43% of global newborn deaths[24]. Moreover, though the global neonatal mortality rate (NMR) has shown a decline from time to time [27], in Ethiopia, the NMR remains unexpectedly high (30 deaths per 1,000 live births) [28]. On the contrary, the United Nations, in collaboration with WHO, targets to reduce neonatal mortality from 20 to 12 per 1000 live births by the end of 2030 and Ethiopia also endorses this target [29], which shows as there is a huge gap to meet the target.

The perisistent challenge in reducing neonatal death rates highlights the need for a system-wide approach to address the main causes of newborn mortality. Hygienic umbilical cord care, aimed at preventing infections that enter the body through the umbilical cord, is a crucial intervention in this effort [2,4,30,31]. Therefore, to scale up hygienic umbilical stump care practices and to reduce neonatal mortality, various strategies, including expanding primary health care services, expanding skilled delivery practices, and promoting essential newborn care practices, have been designed and implemented globally, including in Ethiopia [26,32,33]. Despite these efforts, neonatal mortality rates remain high in Ethiopia, with harmful substances such as butter, animal dung, ash, petroleum jelly, and hair lotion commonly applied to newborns’ umbilical stump [16,28,34]. Existing litratures on this area in Ethiopia has revealed varying results regarding its prevalence, with an increasing trend observed over time [16,17,20] Socio demographic factors educational status of the care giver, age, and maternal health care utilization status were the main determinant factor that influce the practice in Ethiopia [17,35]. The current literature, however, lacks information on the geographical variation and associated factors of harmful umbilical cord stump (UCS) care practices at a national level. Mapping the spatial distribution of harmful UCS care practices in Ethiopia is essential for identifying hotspot and cold spot areas of the practice, enabling policymakers and higher health officials to allocate and mobilize resources more effectively and design targeted interventions to reduce neonatal infections and improve neonatal health outcomes [36,37]. Additionally, the findings from this study will support the country’s efforts in achieving Sustainable Development Goal (SDG 3.2) and contribute to the global body of knowledge on neonatal care practices, offering insights that may be applicable to other low-income countries facing high NMR. In this context, this study aims to assess the burden, spatial distribution, and associated factors of harmful UCS care practices in Ethiopia.

## 2. Methods and materials

### 2.1 Study design, period, and setting

A nationally representative population-based cross-sectional study was conducted in Ethiopia from January 18 to June 27, 2016, using data from the fourth round of the Ethiopian Demographic Health Survey (EDHS, 2016). Ethiopia is the second most populous country in Africa, with a total population of 117,876,226 [38]. The country covers an area of 1,100,000 km^2^ and is situated between latitudes 3° and 15° N and longitudes 33° and 48° E. Ethiopia is divided into nine administrative regions and two administrative cities (Addis Ababa and Dire Dawa). Additionally, it is subdivided into 68 zones, 817 districts, and 16,253 kebeles (the lowest administrative unit). The 2016 survey includes all regions and town administration [28,39].

### 2.2 Source and study population

The source population for this study included all live births within five years preceding the survey in Ethiopia. The study population consisted of all live births within five years preceding the survey in the selected enumeration areas (EAs). Live births in the last five years prior to the 2016 EDHS, with unknown status of harmful UCS care practices, were excluded. Additionally, for the geospatial analysis, clusters with GPS coordinates of zero (0 latitude and 0 longitude) were excluded.

### 2.3 Sample size determination and sampling technique

In the EDHS 2016 survey, a two-stage stratified cluster sampling technique was used to select the final eligible study participants. In the first stage, from a total of 84,915 EAs created during the 2007 Population and Housing Census, 645 enumeration areas (202 from urban areas and 443 from rural areas) were randomly selected using probability proportional to EA size (PPS). A household listing operation was then carried out in all of the selected EAs, which served as the sampling frame for selecting households in the second stage. In the second stage, a fixed number of 28 households per cluster were selected with equal probability using systematic selection from the newly created household listing. Following these procedures, a total of 18,008 households were selected for the survey, of which 15,683 households were interviewed, and a total of 10,641 under-five children were obtained for children survey [39]. When there are two or more under-five children in a household including twins, only the most recent live births (7193) were taken to reduce selection bias and recall bias. In the end, after dropping cases with an "I don’t know" response to the question "Was anything applied to the neonate’s umbilical cord stump after delivery?" a total of 6,775 (a weighted sample of 7,168 live births five years preceding the survey were included in the final analysis. On the other hand, a total of 624 (201 urban and 423 rural clusters) were used for spatial analysis. The detailed procedure regarding the sampling procedure can be obtained from the EDHS, 2016 PDF report [39].

### 2.4 Study variables

The outcome variable for this study was the occurrence of harmful umbilical cord stump (UCS) care practice (yes or no). The independent variables for this study were further classified as individual-level and community-level variables. The individual-level variables were: maternal education, maternal age, maternal marital status, maternal religion, maternal occupation, household wealth status, birth order, number of children, gestational age of the child, size of the child, sex of the child, ANC visit status, place of delivery, media exposure, and distance from the health facility. On the other hand, residence, region, community level of media exposure, and community level of women’s education were the community-level variables for this study. These community-level variables (media exposure, women’s education, and poverty) were constructed by aggregating individual-level factors within each cluster. For the analysis, individual variables were re-categorized, and cross-tabulations with the cluster variable were performed using Stata Version 16 software. The proportion of community-level factors was then computed using Microsoft Excel 2013. Finally, these community-level variables were categorized as high or low status based on the median level value.

### 2.5 Operational definitions

Harmful umbilical cord stump (UCS) care practice is defined as the application of any harmful substance(except 1% chlorohexidine ointment) into the umbilical stump of the neonate, including milk, cow dung, ash, charcoal, dust, hair lotion, baby powder, vaseline, cooking oil, breast milk, and chicken feces [4]. Household wealth status is directly available in the EDHS database which is obtained using principal component analysis and presented with five categories like poorest,v poorer, middle,rich and richest [39]. and it was recategorized into the poor, including poor and poorest, middle, and rich, which includes the richest and richest category. When a woman is single, has not lived with a partner, is not widowed, not divorced, or is no longer living together, her marital status is classified as never in union; otherwise, the mother is classified as in union.

The gestational age of the newborn is obtained from the mother’s perception of the newborn’s total week of gestation and was categorized as preterm, term, and post-term. The size of the child is also obtained from the mother’s perception regarding the weight of her newborn and it was categorized as very large, large, average, small, and very small neonate [39]. Early PNC visit: is the PNC visit with in 0–48 hrs of birth [39].

Household media exposure was obtained by combining whether a respondent reads the newspaper, listens to the radio, and watches television and it was coded as "yes" if the subject was exposed to at least one of the three media, and "no" if the subject was not exposed to at least one of those media [28,39].

### 2.6 Data collection procedures

This study utilized secondary data from the 2016 Ethiopian Demographic and Health Survey (EDHS, 2016). The 2016 EDHS data was accessed from the DHS website after receiving a permission letter through a reasonable online request that explains the overall purpose of the study. The data can be downloaded from the DHS website at http://www.dhsprogram.com. After downloading the data from the above website, the data with the file name “Kids Record (KR)” were used to select relevant data for the study. The data also comprises the Ethiopian ArcGIS shape file which has locations (latitude and longitude coordinates). The GPS takes coordinates for each respondent to represent the clustering of subjects graphically. It is randomly displaced these coordinates by 5 km to protect the confidentiality of respondents.

### 2.7 Data management and analysis

After the extraction of relevant data, further cleaning, coding, and analysis were done using Microsoft Excel 2013 and STATA V.16 software. The sample was weighted before undergoing any statistical analysis.

#### 2.7.1 Statistical analysis

Initially, to get an overall picture of the selected study participants, descriptive statistics were computed using frequency with a percentage and mean with a standard deviation. Given the hierarchical nature of the EDHS data i.e., children are nested in the household, the household is nested in a cluster, fitting a model by ignoring the dependency of observation within the cluster will result in a biased estimate. This implies that the observations with the same cluster will be highly correlated (dependent) which violates the assumption of independence. In this regard, the intra-class correlation (ICC) was calculated to determine the between-cluster variation; an ICC value > 5% indicates that the outcome varied differently between clusters A multilevel binary logistic regression model is fitted to examine the relationship between each independent variable and the outcome variables. First, bi-variable multilevel binary logistic regression models were fitted, and all variables with a p-value less than or equal to equal to 0.25 were candidates for the multivariable model. Then four models comprising different explanatory variables were fitted sequentially. This is a null model without the predictor variable used to estimate the random intercept at the cluster level; Model I includes only individual-level variables; Model II includes only community-level variables, and Model III includes both individual and community-level factors. The log-likelihood ratio (LLR), device, and Akaike information criterion (AIC) were all used to compare the models. Then, the model that has the highest log likelihood or lowest deviance and the highest AIC will be chosen as the best model to estimate the association between independent factors and the outcome variable. The significance of the difference between the models was also assessed using the chi-square likelihood-ratio test at a P-value of 0.01. In addition, the total measures of variation (variation of effect) were assessed using ICC, proportional change in variance (PCV), and the median odds ratio (MOR) [40,41]. he MOR is used to determine the heterogeneity between clusters (the second-level variation) by comparing two persons from two randomly chosen different clusters. Proportional change in variance (PCV) measures the total variation attributed to individual and community-level factors in the multilevel model as compared to the null model. Finally, adjusted odds ratios (AOR) with 95% confidence intervals (CI) were reported in the multi-variable multilevel-logistic regression model and statistical significance was declared at p-value <0.05. The presence of multi-collinearity among independent variables was checked using a variance inflation factor (VIF>10) by running a pseudo-linear regression analysis [42].

#### 2.7.2 Spatial analysis

ArcGIS 10.7.1 software was used for investigating spatial autocorrelation and detection of high and low-risk areas for harmful umbilical cord stump care practices. A spatial autocorrelation (Global Moran’s I) statistic measure was used to assess whether the practice is dispersed, clustered, or randomly distributed [43]. Moran’s I values close to -1 indicate a dispersed pattern, 0 indicates random, pattern and close to a+ 1 indicates clustering of harmful US care practice in Ethiopia. The maximum peak distance at which harmful UCS care practice becomes more prominent was determined using incremental spatial autocorrelation.

### I. Hot-spot and cold-spot analysis

The Getis-Ord Gi* hot spot analysis was used to identify spatial clusters of high values (hot spots) and spatial clusters of low values (cold spots). The hot spot areas indicated that there is a high proportion of harmful UCS care practice and the cold spots indicated that there is a low proportion of harmful UCS care practice.

### II. Ordinary kringing interpolation analysis

A spatisl spatial interpolation technique was used to predict harmful UCS care practice in the un-sampled areas of the country based on sampled EAs using ordinary Kriging spatial interpolation methods.

### III. SaTScan analsyis

In the presence of clustering of harmful UCS care practices, a Bernoulli probability-based model of spatial scan statistics was employed using Kuldorff’s SaTScan version 9.6 software to determine the purely statistically significant spatial clusters [44]. The scanning window that moves with 1 kilometer (km) radius across the study area, in which children with harmful US care were considered cases and those with no harmful UCS care were considered non-cases (controls), was fitted to run the Bernoulli probability model. The number of cases in each location had a Bernoulli distribution, and the model required data for cases, controls, and geographic coordinates. The default maximum spatial cluster size of 50% of the population was used as an upper limit, which allows both small and large clusters to be detected and ignores clusters that contain more than the maximum limit. For each potential cluster, the likelihood ratio test statistic was used to determine if the observed harmful UCS care within the cluster was significantly higher than expected or not. The primary, secondary, and tertiary clusters were identified, assigned p-values, and ranked based on their likelihood ratio test, based on 999 Monte Carlo replications.

### 2.8. Ethical considerations

Ethical clearance was obtained from the Institutional Review Board (IRB) of Bahir Dar University, College of Medicine and Health Sciences, with protocol number 601/2022. Since the study used secondary data (EDHS 2016 survey), informed consent was not required for this analysis. However, permission to access the DHS data was obtained from the primary DHS data archivist through an online request via http://www.dhsprogram.com (**S1 File**). The survey it self is conducted by The survey itself was conducted by the Central Statistical Agency (CSA) of Ethiopia in collaboration with local government, nongovernmental, and international development partners, following the required ethical procedures. In the DHS data, there are no names of individuals or household addresses. In addition, the data were treated as confidential, and no attempt should be made to identify any household or individual respondent interviewed in the survey. Finally, the Information retrieved was used only for statistical reporting and analysis of our registered research.

## 3. Result

### 3.1 Individual and community level socio-demographic variables

A total of 7,168 (100%) newborns were included in this study. Regarding the socio-demographic profiles of the participants, the majority lived in rural areas (6,357 or 88.7%), were married (6,629 or 93.0%), and did not attend formal education (4,602 or 64.2%). Concerning community-level variables, more than half (3,718 or 51.9%) of the respondents were from communities with low educational status. Additionally, nearly four in ten (2,835 or 39.5%) of the respondents came from communities with a high poverty level **(Table 1).**

**Table 1:** Socio-demographic and economic characteristics of the study participants of harmful US care practice in Ethiopia using EDHS 2016 data, 2023.

Variables (N = 7168)	Category	Harmful US care Practice		Frequecy (%)
		Yes = 1082 (15.09%)	No = 4325(84.01%)	
Head of the HHs	Male	961	5,173	6,134 (85.6%)
	Female	121	913	1,034 (14.4%)
Age category	Age 15–24	218	1,474	1,692(23.6%)
	Age 25–34	417	3,195	3,612 (50.4%)
	Age 35 and above	447	1,417	1,864(26%)
Religion	Orthodox	456	2,174	2,650 (37.0%)
	Moslem	317	2,380	2,697 (37.6%)
	Protestant	235	1,355	1,590(22.2%)
	Others*	53.	177	230(3.2%)
Marital status	Married/ in union	987	5,642	6,629(92.5%)
	Not_in union	95	444	539(7.5%)
Educational status of the mother	No formal education	671	3,932	4,603 (64.1%
	Primary education	345	1,672	2,017 (28.1%)
	Secondary	44	320	364(5.2%)
	Tertiary and above	22	162	184 (2.6%)
Educational status of the husband	No formal education	523	2,712	3,235 (48.2%)
	Primary education	397	2,203	2,600 (38.8%)
	Secondary	81	475	556 (8.2%)
	Tertiary and above	22	298	320 (4.8%)
Working status of the Respondent	Working	789	4,357	5,146 (71.8)
	Not working	263	1,729	2022 (28.2%)
Region	Tigray	124	365	489 (6.8%)
	Afar	5	61	66 (0.94%)
	Amhara	244	1,262	1,506(21.0%)
	Oromia	397	2,615	3,012 (42.0%)
	Somali	47	216	263 (4.0%)
	Benishangul	7	73	80(1.1%)
	SNNPR	243	1,292	1,53 (21.4%)
	Gambela	2	18	20(0.3%)
	Harari	3	13	16(0.2%)
	Addis Abeba	7	143	150(2.1%
	Dire Dewa	3	28	31(31%)
Residence	Urban	73	738	811 (11.3%)
	Rural	1009	5,348	6,357 (88.7%)
Health_insurance	Enrolled	53	231	284 (4.0%)
	Not_enrolled	1029	5,855	6884 (96.0%)
Household Wealth_index	Poor	508	2,736	3,244 (45.2%)
	Middle	242	1,275	1,518 (21.2%)
	Rich	332	2,075	2,407 (33.6%)
Household media_exposure	Not exposed	732	4,042	4,774 (66.6%)
	Exposed	350	2,044	2,394 (33.4%)
Community poverty–level	Poor	456	2,379	2,835 (39.5%)
	Rich	626 .	3,707	4,333 (60.5%)
Community educational- status	Low	638	3,779	4,417 (61.6%)
	High	444	2,307	2,751 (38.4%)
Distance to Health facility	Big problem	687	3,707	4,394 (61.3%)
	Not big problem	395	2,379	2,774 (38.7%)
Community media_exposure	Low	709	1881	4,590 (64.0%)
	High	373	2205	2,578 (36.0%)

IQR = Inter-quartile range, HH- Households, Others- catholic, traditional.

### 3.2 Maternal and child health characteristics of the study participants

In this survey, In this study, more than two-thirds of the mothers (5,018 or 70%) gave birth at home. Additionally, only 60.8% of pregnant mothers in the last five had attended antenatal care (ANC) follow-up, and 40.9% took iron and folic acid (IFA) tablets. Of the total recent live births, 3,730 (52%) were male, and 2,900 (40.5%) had a normal birth weight. Regarding postnatal care services, only 422 (6.0%) of the study participants received early postnatal care within two days of discharge from a health facility (**Table 2).**

**Table 2:** Maternal and child health characteristics result of the study participants of harmful UCS care practice in Ethiopia, 2023.

Variables (N = 7168)	Category	Harmful umblical cord care Practice		Frequency (%)
		Yes = 1082 (15.09%)	No = 4325(84.01%)	
ANC visit	Yes	624	3,745	4,369 (61.0%)
	No	458	2341	2,799 (39.1%)
ANC Visit	No	458	2341	2,799 (39.1%)
	ANC1to 3	381	1949	2330 (32.5%)
	≥ ANC4	243	1,796	2,039 (28.5%)
IFA intake status	Yes	407	2,434	2,841 (40.9%)
	No	674	3,652	4,327(59.1%)
Delivery place	Health institution	254	1,896	2,150 (30.0%)
	Home	828	4,190	5,018 (70.0%)
Birth order	1^st^	185	1,127	1,312 (18.8%)
	2^nd^ -3^rd^	317	1,814	2,131 (29.7%)
	Four and above	580	3,145	3,725 (60.0%)
Total living children	0	4	44	48 (0.7%)
	1–3	556	3,162	3,717 (51.9%)
	≥ 04	522	2,881	3,403 (47.4%)
Sex of the neontate	Male	525	3,205	3,730 (52%)
	Female	557	2,881	3,438 (48%)
Birth_weight	Large birthweight	322	1934	2256 (31.5%)
	Normal	425	2,476	2,901 (40.5%)
	LBW	335	1,676	2,011 (28.0%)
Early PNC visit	Yes	54	366	420 (6.0%)
	No	1028	5,720	6,748 (94.0%)

Others- Partner/ husband, mother in law, father in law, someone else.

IFA- Iron Folic Acid intake.

### 3.3 Magnitude of harmful UCS care practice in Ethiopia

**Fig 1:**
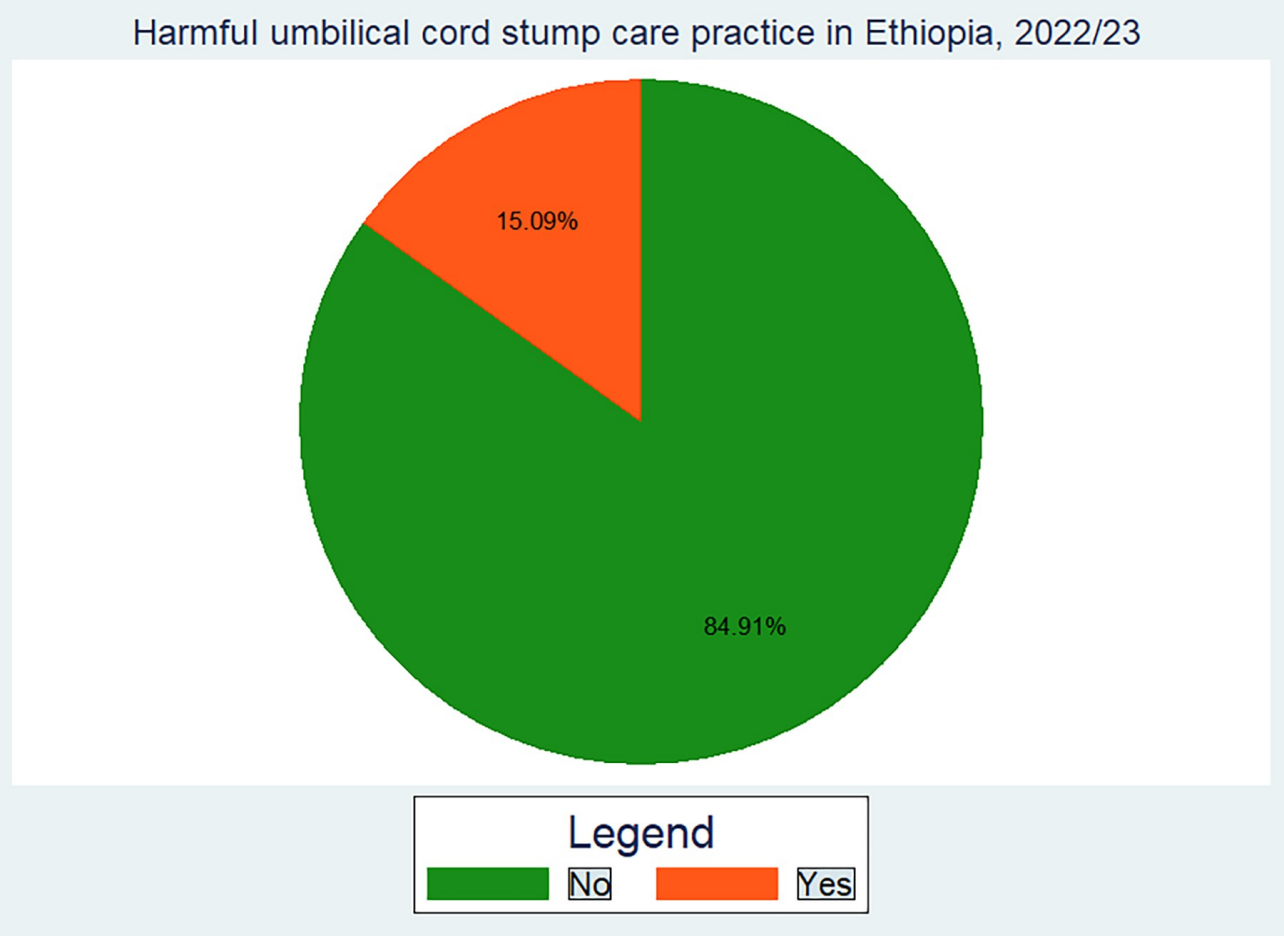
Harmful umblical cord stump care practice status in Ethiopia, 2023.

The prevalence of harmful UCS care practices in Ethiopia was estimated to be 15.09% (95% CI: 13.9–16.3) **(Fig 1)**. Oil was the most commonly used harmful substance (744.4 (68.4%)), followed by butter (15.4%), ointment (14.1%), ash (3%), and cow dung (4.7%). Of the 1082 pregnant women who applied harmful substances to the neonate’s umbilical cord stump, 777 (72%) had at least one ANC visit. Indeed, the proportion of harmful UCS are practices was higher in female neonates (15.9) than males (14.4) **(Fig 1).**

### 3.4 Spatial distribution of harmful UCS care practice in Ethiopia

**Fig 2:**
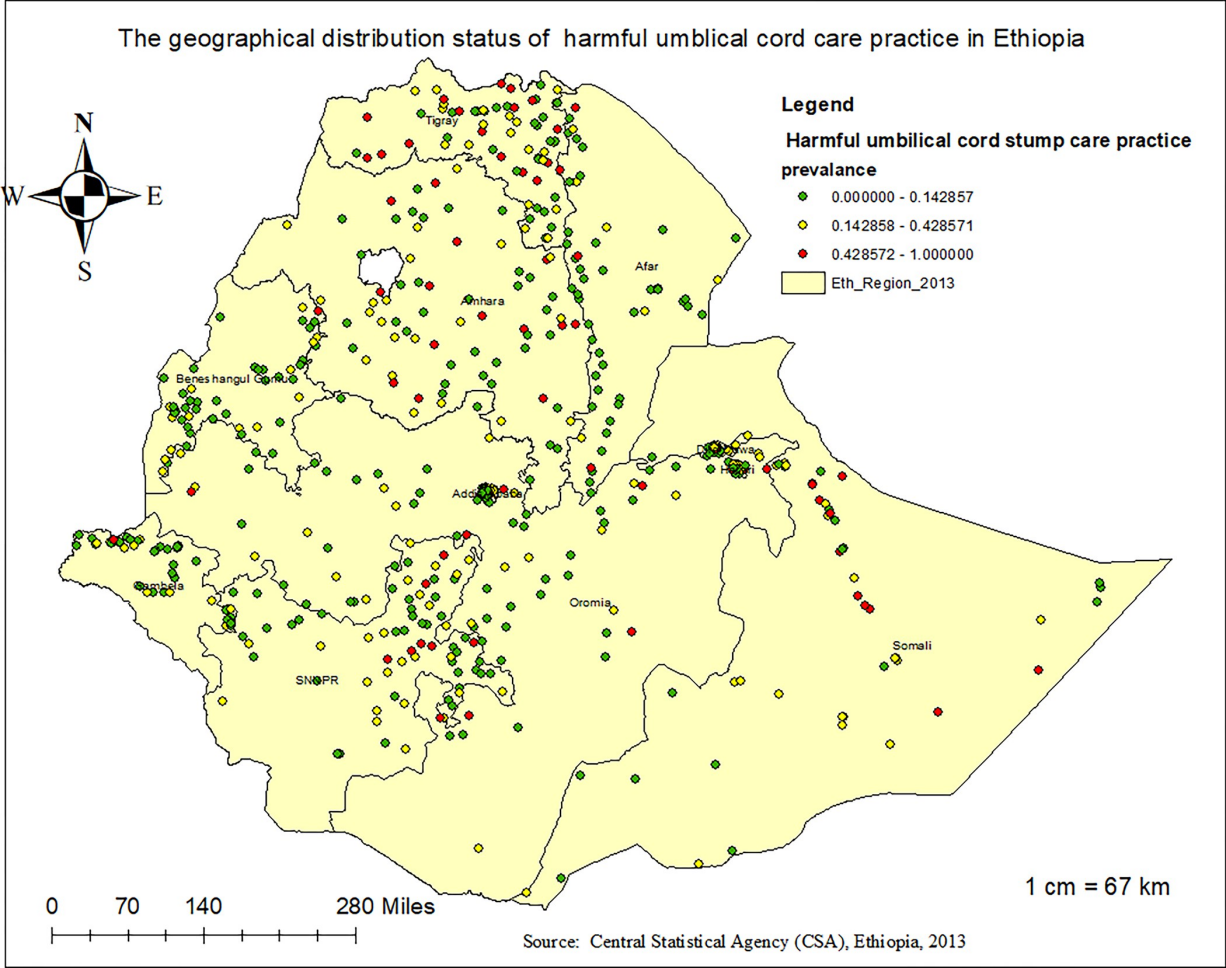
Spatial distribution status of harmful UCS care practice in Ethiopia, 2023.

A total of 624 clusters were used to run the spatial analysis of harmful UCS care practices in Ethiopia. Each point on the map corresponds to an enumeration area, showing the proportion of harmful UCS care practices within each cluster. The red points on the map indicate areas with a high proportion, while the green points indicate areas with a low proportion of harmful UCS care practices in Ethiopia. in this context, a high prevalence of such care practices was observed in northern Ethiopia (Tigray and Amhara regions), eastern Ethiopia (Somali, Dire Dawa, and Harari regions), and the SNNPR region, ranging from 42.8572% to 100%. In contrast, a low prevalence of harmful UCS care was detected in the Addis Ababa and Benishangul-Gumuz regions **(Fig 2)**.

#### 3.4.1 Spatial and Incremental autocorrelation result of harmful UCS care practice

**Fig 3:**
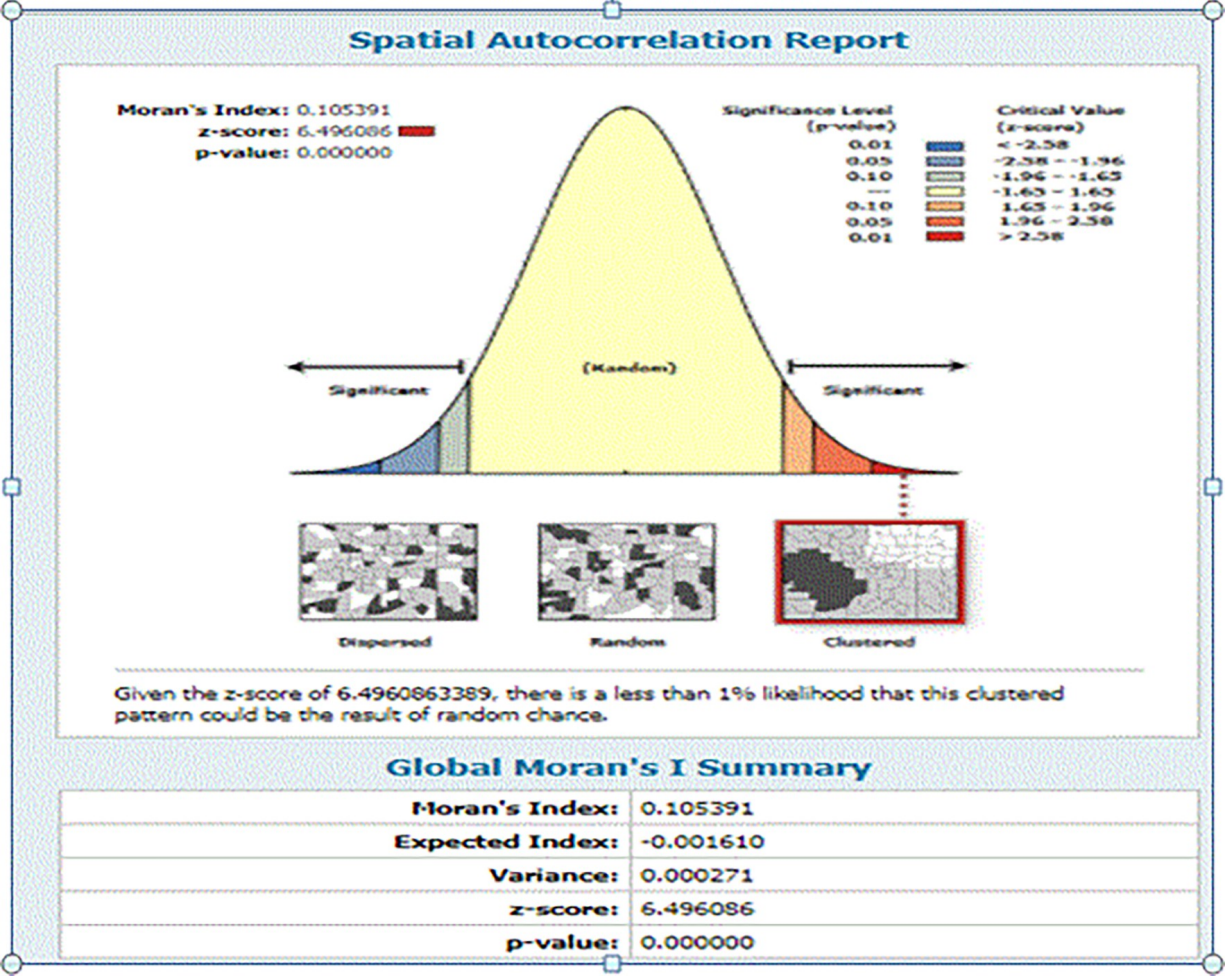
Spatial autocorrelation analysis result of harmful UCS care practice in Ethiopia, 2023.

**Fig 4:**
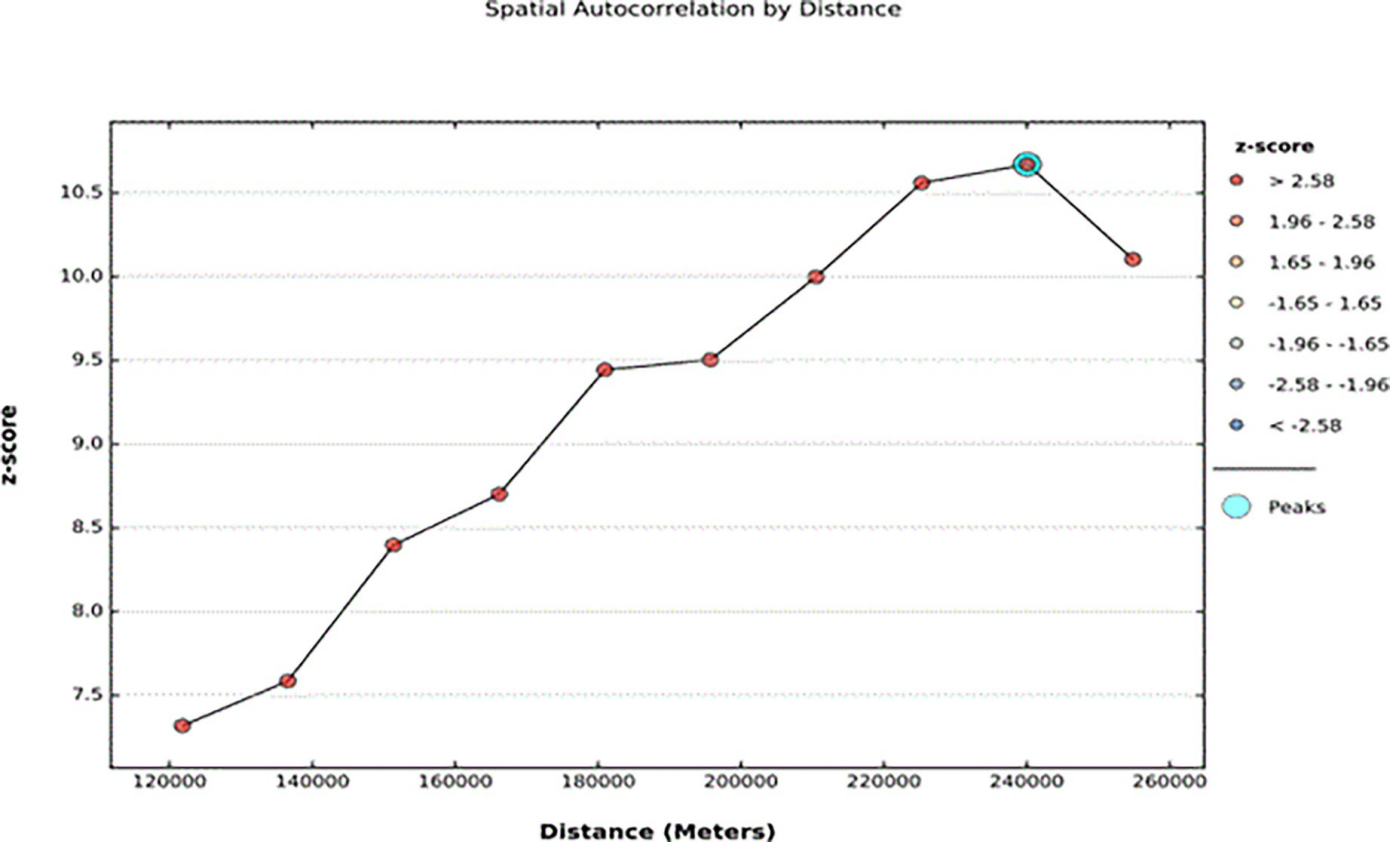
The spatial incremental autocorrelation analysis result of harmful UCS care in Ethiopia, 2023.

In the 2016 EDHS survey, the spatial distribution of harmful UCS care practices was clustered in specific geographical areas of Ethiopia. The global Moran’s I value was 0.105391 (Z-score = 6.496086, P-value < 0.01), indicating that the the practice showed a clustered patternof distribution in Ethiopia, with less than a 1% likelihood that this pattern occurred by chance. The bright red and blue colors at the ends of the spectrum indicated an increased significance level in the distribution of harmful UCS care practices **(Fig 3).** The incremental spatial autocorrelation for a series of distances presented by a line graph with a corresponding z-score was computed to determine the average nearest neighbor and the minimum and maximum distance bands at which the spatial process promoting clusters is most pronounced. A total of 10 distance bands were detected at a beginning distance of 121803.00 metres, and the first maximum peak (clustering) was observed at 240061.63 metres (**(Fig 4)**.

#### 3.4.2 Hot Spot Analysis (Getis-OrdGi Statistic) result of harmful UCS care practice

**Fig 5:**
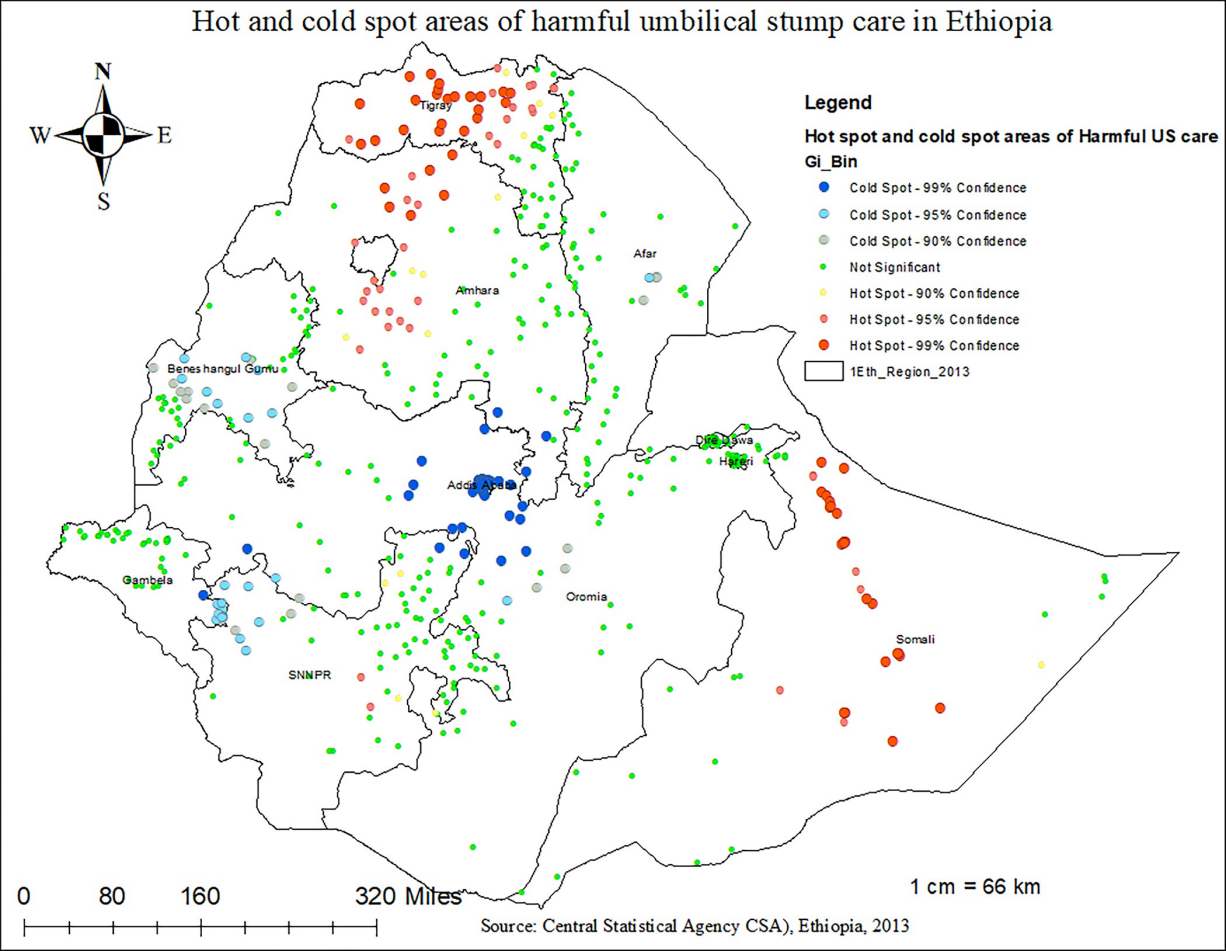
Hot spot areas analysis result of harmful UCS care practice in Ethiopia, 2023.

**Fig 6:**
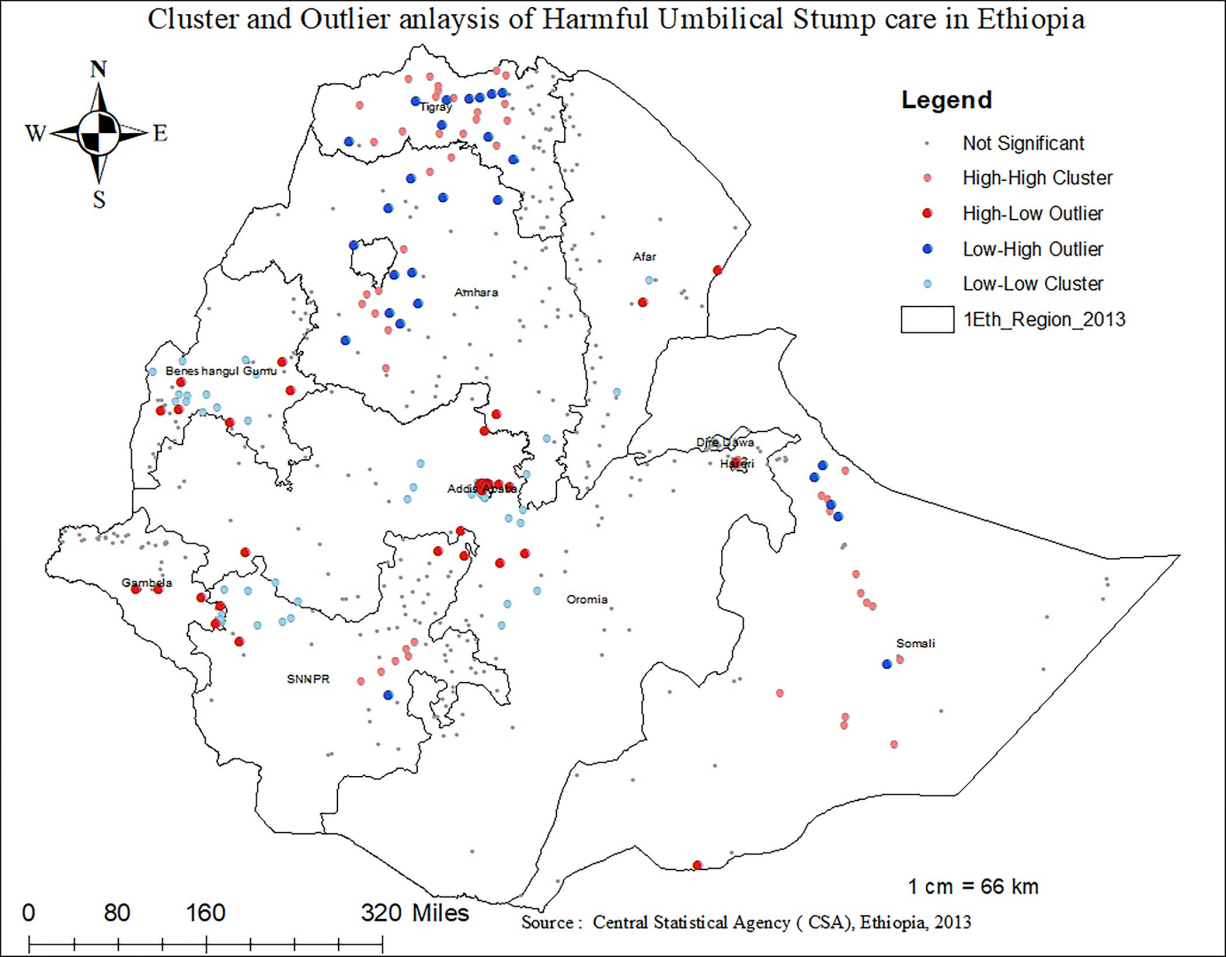
Cluster and outlier analysis result of harmful UCS care in Ethiopia, 2023.

The Getis-OrdGi statistics showed that the hotspot areas (red-colours) of harmful UCS in Ethiopia were mainly observed in the Tigray, Somal, and Amhara regions. On the other hand, clusters located in Addis Abeba, Beninshagul-Gumez, and the western part of SNNPR were found to be the cold-spot areas of UCS practice **(Fig 5).** Indeed, the Anselin Local Moran’s I analysis of harmful UCS care practice revealed that there were significant outliers. Accordingly, high outliers for harmful UCS care practice were detected in Gambella, Benishangul Gumez, Tigray, and the central part of Oromia regions. Besides, low outliers of harmful UCS care practice are detected in Tigray, Amhara, and the eastern part of Somalia in Ethiopia **(Fig 6)**

#### 3.4.3 The interpolation method of harmful US care practice

**Fig 7:**
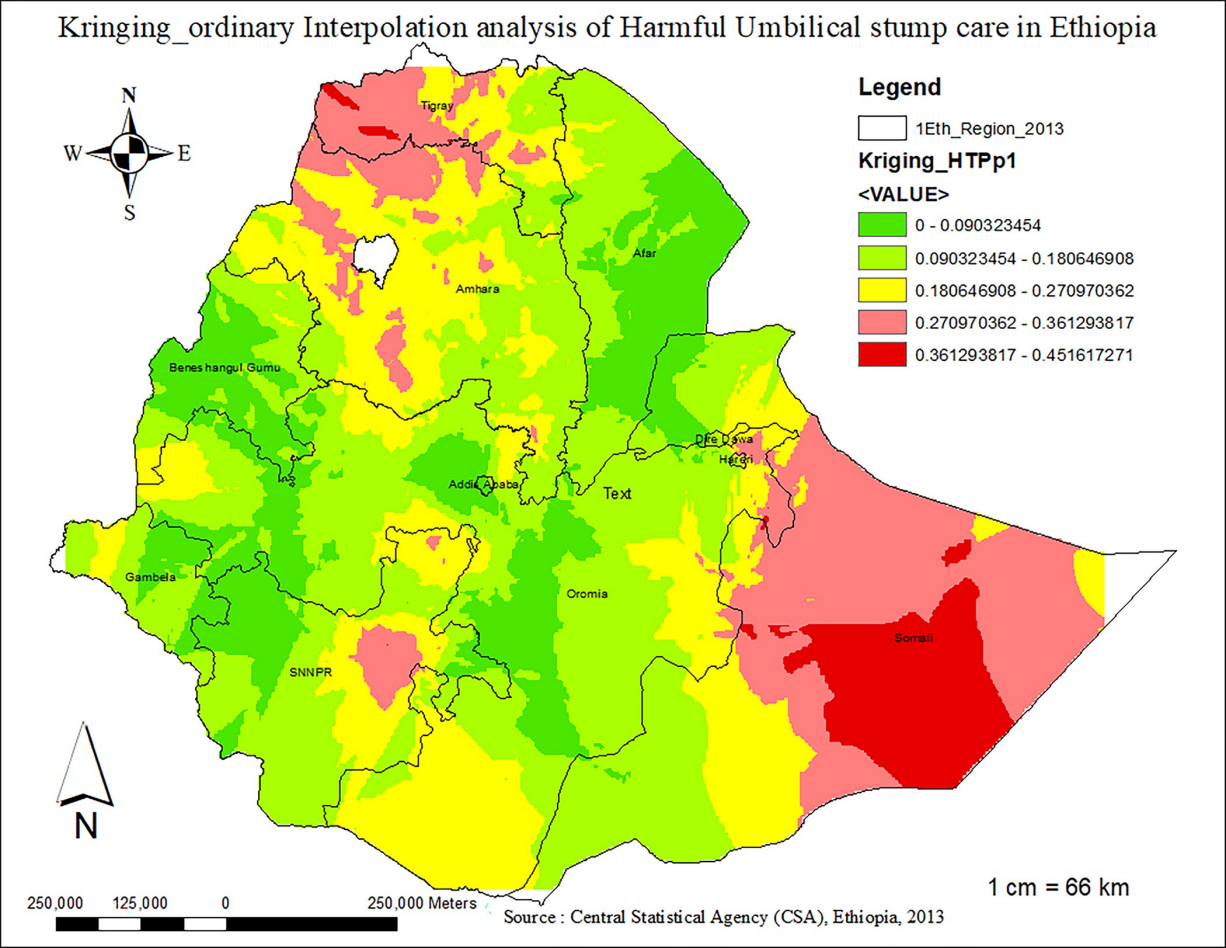
. The geospatial kriging interpolation prediction graph of harmful UCS care practice in Ethiopia, 2023.

In order to predict the burden of harmful UCS care practices in unsampled areas of Ethiopia, we used ordinary kriging georeferenced statistical interpolation anlaysis method. Based on the geostatistical kriging-ordinary interpolation analysis results, the probability of harmful umbilical cord care practices increased as we moved from the red- to the green-colored areas. Consequently, the Somali Regional State, central and northern Tigray, parts of the Amhara region, and the northern part of the SNNPR region had a high prevalence of harmful umbilical cord stump care practices, ranging from 27% to 45.17% **(Fig 7).**

#### 3.4.4 SaTScan spatial analysis result of harmful UCS care practice in Ethiopia

**Fig 8:**
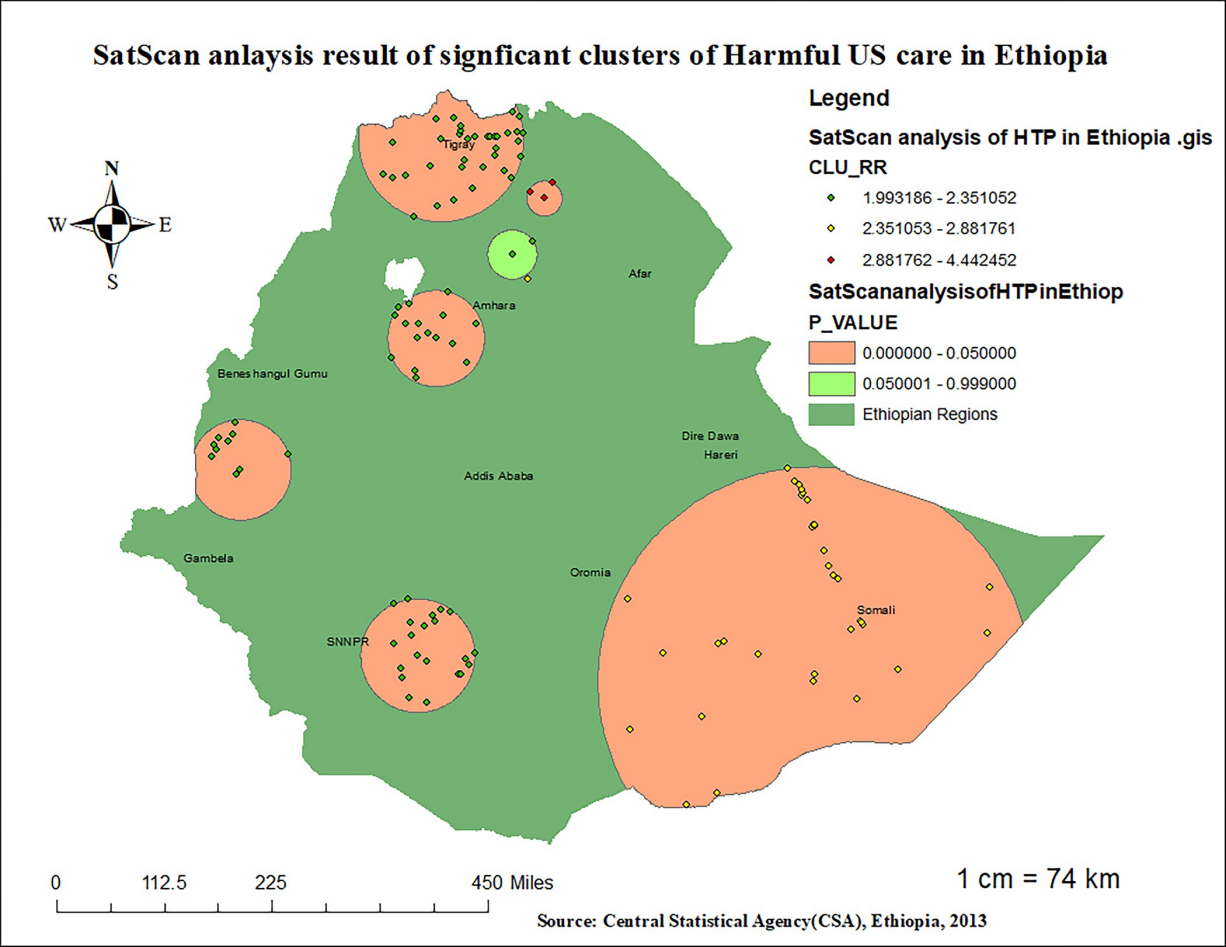
SaTScan HotSpot analysis result of harmful US care practice in Ethiopia, 2023.

As presented in Fig 7 and Table 3 below, the SaTScan spatial analysis detected a total of 120 statistically significant SaTScan clusters of harmful UCS care practice in Ethiopia. This implies that the burden of harmful UCS care practice in Ethiopia was higher inside the SaTScan circular window compared to outside the SaTScan window. The most likely primary SaTScan clusters of harmful US care practice were observed in the SNNPR region, particularly in Wolita, Gamo-Gofa, and sidama zones (coordinates = 6.266023 N, 37.529041 E, radius = 95.67 km, LLR = 53.671268,RR = 2.35, P-value< 0.001). Neonates who were born in this spatial window had 2.35 times more risk of having harmful umbilical cord care compared to those neonates born outside the spatial window. The most likely secondary SaTScan clusters were identified in zones of the Tigray region (coordinates = 5.848373 N, 43.527981 E) / 361.54 km, LLR = 31.898720, RR = 2.88, p <0.01). Moreover, the third, fourth, and fifth most likely clusters of HS care practise were detected in Tigray and Amhara regions, respectively **(Fig 8, Table 4).**

**Table 3:** Multi-variable multilevel binary logistic regression analysis result of factors associated harmful UCS care practice in Ethiopia, 2023.

Community and individual level factors	Models			
	Null model AOR (95%CI)	Model I (AOR 95%CI)	Model II (AOR 95%CI)	Model III (AOR 95%CI)
Respondents age				
		**1.07 (1.02 1.12)**		**1.07 (1.02 1.12)**
Household head Male Female				
		1		1
		0.77(0.53 1.12)		1.31(1.04 1.67)
Household wealth index poor Middle rich				
		1		1
		1.01 (.64 1.61)		0.92(0.58 1.46)
		0.84 (0.59 1.20)		0.83 (0.57 1.22)
Delivery Home Health institution				
		1		1
		**0.61 (0 .41 0.91)**		**0.64 (0.42 0.97**)
Birth order				
		0.91(0.82 1.01)		**0.90 (0.81 0 .99)**
Sex of neonate Male Female				
		1		1
		**1.31 (1.03 1.66)**		**1.31(1.04 1.67)**
PNC visit No Yes				
		1		1
		0.76 (0.46 125)		0.75 (0.45 1.23)
Birth weight Large Normal Small				
		1		
		1.07 (0.80 1.44)		1.05 (0.75 1.45)
		1.19 (.86 1.64)		1.24 (0.84 1.84)
Community Level factors of harmful US care practice				
Residence Urban Rural				
		
			**2.54 (1.24 5.17)**	**2.18 (1.05 4.52)**
Region Addis Abeba Tigray Afar Amhara Oromia Somalia Benishangul SNNPR Gambela Harari Dire Dewa				
			**1**	**1**
			**3.17 (1.16 8.66)**	**3.79 (1.38 9.38)**
			0.75 (.25 2.24)	0.91 (0 .29 2.86)
			1.78 (.63 5.09)	1.66 (0.58 4.78)
			1.07 (0.37 3.15)	1.2 (0.39 3.63)
			2.41 (0 .87 6.70)	**2.95 (1.02 8.52)**
			0.75 (.25 2.22)	0.88 (0.29 2.67)
			1.52(0.52 4.45)	1.63 (0.55 4.84)
			1.06 (0.39 2.85)	1.23 (0.45 3.42)
			**2.85(1.07 7.59)**	**3.51 (1.28 9.60)**
			1.51(0 .55 4.16)	1.85 (0.66 1.58)
Distance to Health facility Big problem Not Big problem				
			1	1
			0.84 (0.61 1.15)	0.89 (0.65 1.22)
Community Media-exposure Low High				
			1	1
			1.13 (0.72 1.78)	1.22(0.77 1.92)
Community poverty level poor rich				
			1	1
			0.89 (0.55 1.43)	0.88 (.54 1.45)
	**Measure of random effect (variation)**			
Variance	2.486172	2.4465	2.296874	2.288999
ICC (%)	**43.07%**	42.67%	41.13%	40.0%
PVC (%)	Reference	1.56%	7.6%	8.6%
MOR	4.07	4.04	3.91	3.9
	Model fitness			
Log likelihood	-2599.4548	-2534.038	- 2578.4627	**-2516.9472**
Deviance	5,198.9096	5,068.076	5,156.9254	**5,033.8944**
AIC	5202.91	5092.076	5188.925	**5085.894**

1- Reference category, AOR-adjusted odds ratio, CI- confidence interval, Bold letter—statistically significant variables in the full model.

**Table 4:** The most likely SatScan clusters of harmful UCS care practice in Ethiopia, 2023.

Year	Cluster	Area of clusters	Coordinate /radius	Popul ation	Case	RR	LLR	P-Value
2016 EDHS	Primary clusters	505, 503, 450, 434, 406, 86, 180, 141, 20, 53, 342, 574, 182, 32, 50, 162, 232, 347, 113, 600, 306	(6.266023 N, 37.529041 E) / 95.67 km	529	171	2.35	53.671268	< 0.01
	Secondary clusters	164, 358, 85, 138, 278, 492, 92, 543, 490, 146, 318, 187, 171, 198, 95, 556, 497, 520, 480, 521, 588, 553, 458, 208, 77, 214, 251, 394, 573, 239, 116, 629, 22, 286, 568	(5.848373 N, 43.527981 E) / 361.54 km	160	67	2.88	31.898720	<0.01
	3^rd^ Clusters	583, 98, 268, 255, 528, 78, 181, 258, 340, 584, 188, 597, 253, 400, 612, 551, 590, 636, 156, 81, 425, 296, 80, 84, 322, 504, 579, 479, 45,481, 575, 355, 638, 89, 461	(14.085315 N, 37.856586 E) / 138.18 km	378	117	2.17	31.898720	<0.01
	4^th^ Clusters	160, 424, 384	(13.197265 N, 39.457916 E) / 29.66 km	44	29	4.44	29.226909	<0.01
	5^th^ Cluster	24, 403, 120, 429, 167, 456, 382, 375, 206, 38, 73, 474, 516, 3, 132,431	(11.074276 N, 37.790726 E) / 81.07 km	358	105	2.04	24.600331	<0.01
	6^th^ Cluster	175, 248, 462, 193, 643, 275, 374, 395, 17, 304	(9.060100 N, 34.818806 E) / 84.43 km	145	50	2.34	16.990590	<0.01

RR- Relative Risk, LLR- log-likelihood ratio.

### 3.5 Multilevel mixed-effect binary logistic regression analysis

#### 3.5.1. Random effect estimation

As indicated in Table 4, the results of the intraclass correlation coefficient (ICC) in the empty model showed that **43.07%** of the total variation in harmful UCS care practices was attributed to differences between communities or clusters. Therefore, fitting a multilevel (two-level) binary logistic regression model that accounts for the dependency or correlation of observations within clusters is reasonable. In the multilevel binary logistic regression analysis, the full model (Model III), which included both individual- and community-level variables, was selected as the best-fitting model because it had the lowest deviance and AIC value (**deviance = 5,033.8944, AIC = 5085.894**). The presence of heterogeneity between clusters was also explained by the median odds ratio (MOR). The MOR in the full model was **3.90,** implying that the odd’s of harmful UCS care practice is 3.90 times higher among newborn’s from communities with a high risk versus a low risk of the practice. Moreover, the proportional change in variance (PCV) in the final model also indicates that 8.6 percent of the variation in harmful UCS care in the community was explained by both communityand individual-level variables.

#### 3.5.2 Fixed effect statistical analysis result

n the bivariable multilevel logistic regression analysis, the following variables were selected as candidates for the multivariable multilevel logistic regression model at a p-value < 0.25: respondent’s age, neonate sex, place of delivery, postnatal care, household economic status, birthweight, household head, residence, distance to a health facility, community poverty level, community media exposure status, and region. In the final model, respondent’s age, place of delivery, sex of the neonate, birth order, region, and residence were significantly associated with harmful UCS care practices in Ethiopia. When the age of the mother increases by one unit, the odds of harmful UCS care practices increase by 7% (AOR = 1.07, 95%CI 1.02–1.12). The practice of applying harmful substances to the umbilical cord stump of neonates delivered at a health institution was 36% less likely (AOR = 0.64, 95% CI: 0.42–0.97) to neonates delivered at home. Compared to male neonates, female neonates were 1.31 times more at risk of experiencing harmful UCS care (AOR = 1.31, 95% CI: 1.04–1.61). The odd’s of applying a harmful substance to the Umblical Stump of neonates was 2.18 (AOR = 2.18, 95%CI: 1.05–4.52) times higher among mothers of rural dwellers than urban dwellers. Additionally, when compared to respondents from Addis Ababa, mothers from Tigray, Somali, and Harari regions were 3.79 times (AOR = 3.79, 95% CI: 1.38–9.38), 2.95 times (AOR = 2.95, 95% CI: 1.02–8.52), and 3.51 times (AOR = 3.51, 95% CI: 1.28–9.60) more likely to apply harmful substances to their neonate’s umbilical cord stump, respectively. Finally, the odds of harmful umbilical cord stump care practices decreased by 10% (AOR = 0.90, 95% CI: 0.81–0.99) with a one unit increase in the child’s birth order **(Table 4)**.

## 4. Discussion

Harmful or unhygienic UCS care is a major public health problem, as it serves as a primary pathway for bacteria to enter the neonate’s body, potentially causing local and severe infections, sepsis, and ultimately death [4,13,22]. This study revealed that the magnitude of harmful umbilical cord care practices was 15.09% (95% CI: 13.9–16.3). This prevalence is consistent with a report from Mandura District, northwest Ethiopia [45]. However, it is significantly lower than findings from studies conducted at Mizan-Tepi University Teaching Hospital (western Ethiopia), public hospitals in eastern Ethiopia, Kenya, Rwanda, Nigeria, and South Asian countries [13,15,17,20,21,46,47]. This variation may be due to differences in study settings (the two Ethiopian studies were conducted in health institutions where only sick neonates are addressed) [17,20], study period and cultural values and beliefs across countries.

The practice of harmful umbilical cord care was common in the Tigray, Somali, Harari, Amhara, and SNNPR regions of Ethiopia. The odds of harmful umbilical cord care practices are higher among neonates from Tigray, Somali, and Harari regions compared to those from Addis Ababa. In line with this observation, the spatial analysis in this study identified that the geographical distribution of harmful UCS care is non-random and highly clustered (Z-score = 6.496086, P-value < 0.01). Hotspot areas of harmful UCS care practice in Ethiopia were primarily observed in the Tigray, Amhara, and Somali regions. Conversely, clusters in Addis Ababa, Benishangul-Gumuz, and the western part of SNNPR were found to be cold-spot areas with lower prevalence of harmful UCS practices. Indeed, a total of approximately 120 clusters in SNNPR, Tigray, Amhara, and Somali regions were identified as significant clusters of harmful UCS care practice in Ethiopia. The highly clustered nature of harmful UCS care practices in these areas can be attributed to deeply rooted socio-cultural, religious, and economic perspectives that influence whether communities engage in such practices [48–50]. Besides, The lower burden of harmful UCS care practices in Addis Ababa compared to other regions might be due to higher educational levels among mothers, increased awareness and knowledge regarding essential newborn care, and relatively better accessibility to health services in Addis Ababa [39,51,52]. In line to this, it is also observed that the odds of applying harmful substances on the umblical cord of the neonate was 2.18 (AOR 2.18, 95%CI: 1.05–4.52) times more likely among neonates from rural residence compared to the urban dwellers. This finding is similar with studies conducted in Pakistan and Ethiopia [45,53]. The observed link between residence and harmful UCS care practice might be secondary to the easy accessibility of health services and health-related information and the high educational status of women in urban areas compared to rural residents [48,54]. This further indicates that improved accessibility to health services is associated with a reduced likelihood of harmful umbilical cord care practices, which could significantly reduce neonatal mortality.

In this study, harmful UCS care practices decreased by 36% among neonates delivered in health institutions compared to those delivered at home. This finding is consistent with prior studies conducted in Ethiopia and Nigeria [14,35]. The observed association may be due to the fact that women who deliver at health institutions have more opportunities to receive comprehensive information and counseling about recommended newborn practices and the potential risks of applying harmful substances to the umbilical stump of their neonate. In contrast, mothers who deliver at home are more likely to be influenced by community cultural values and practices that encourage such a harmful practices. Additionally, women who give birth in health institutions are likely to be more educated and have attended more antenatal care (ANC) visits, which indirectly increases their knowledge of appropriate umbilical cord stump care [55,56]. This implies that comprehensive strategies and activities need to be designed and implemented to decrease the prevalence of home deliveries.

In the current study, it was observed that as maternal age increased, the likelihood of using unhygienic substances on the neonate’s umbilical cord also increased. This finding is consistent with a previous literature review study conducted in Ethiopia [35]. A possible explanation is that older mothers are more likely to adhere to and promote traditional cultural beliefs and practices related to newborn care compared to their conterparts [57]. On the other side, this finding is contrasts with a study conducted in Nigeria [58],which suggests that the experience get over time may lay a valuable lessons to the womnes to reduce the practices.

The sex of the neonate was also an other factor associated with harmful UCS care practices in Ethiopia. Female neonates were 1.31 times more likely to be exposed to to the practice compared to counterparts. This finding is supported by prior cross-sectiona research conducted at the University of Benin Teaching Hospital, Nigeria [59,60]. The observed link might be due to the fact that, particularly in low-income countries, there is a strong traditional preference for sons, which favors male children for beneficial care over female children [60,61]. This preference might initially result in the application of unfamiliar traditional practices on female children, with a potential shift towards male children as families appreciate the adavantage and disadvantage of these practices. On the other hand, another study in Sokoto district,Nigeria [14] showed that the neonate sex had no significant relationship with umbilical cord care practices, which might be related with the socio-demographic descripancies of the mothers.

Finally, this study also found that harmful umbilical cord care practices decreased by 10% with each unit increase in the birth order of the neonate. This can be attributed to the fact that mothers experiencing their first birth often lack information and experience regarding beneficial umbilical cord care practices and may rely heavily on decisions made by their mother-in-law, father-in-law, and other family members concerning neonatal and childhood care. This further implies that primiparous mothers may not have adequate experience and knowledge of recommended and essential newborn care practices, making their newborns more likely to be exposed to harmful traditional practices, such as unhygienic umbilical cord care. In contrast, a related study conducted in southwest Ethiopia revealed that essential newborn care practices were significantly higher among first-order neonates compared to higher-order neonates [62]. The possible reason for the observed discrepancy could be described as families in this community might give special value to the first child, and this value goes down as the number of live children increases, sometimes because of saturtation of their family needs.

### 4.1 Strength and limitation of the study

The major strength of this study is the use of a large and nationally representative dataset. Additionally, a multilevel fixed-effects regression model was employed to account for the correlation of observations within clusters and to investigate the impact of secondary-level variables on harmful umbilical cord care practices in Ethiopia. However, the study has some limitations that should be considered when interpreting the results. First, the effects of important behavioral and attitudinal factors were not investigated. Second, due to the retrospective nature of the data, recall bias may have been introduced. Moreover, aggregating individual-level variables to determine community status can introduce drawbacks such as ecological fallacy and the obscuring of individual-level variations.

## 5. Conclusion and recommendation

In Ethiopia, the distribution of harmful umbilical cord stump (UCS) care practices is non-random and highly clustered in the SNNPR, Somalia, Tigray, and Amhara regions. Both individual-level factors (such as maternal age, respondent’s age, place of delivery, and sex of the neonate) and community-level factors (such as residence and region) were found to be significantly associated with harmful UCS care practices in Ethiopia. The Ministry of Health in Ethiopia and other stakeholders need to focus on neonates born in these identified hotspot areas. Key interventions should include improving maternal health service utilization and targeting older mothers and female neonates. Additionally, further mixed researches need to be undertaken to determine the current status of these practices and to explore the cultural values, beliefs, and attitudes related to the practice.

## Supporting information

S1 Checklist STROBE 2007 (v4) checklist of items to be included in reports of observational studies in epidemiology*. (DOC)

S1 File (DTA)

## Abbreviations

AIC: Akakies Information Criteria
AOR: Adjusted Odds Ratio
EA: Enumeration Area
EDHS: Ethiopian Demographic and Health Survey
ICC: Intra cluster correlation
IQR: Inter Quartile Range
MOR: Median Odds Ratio
PCV: Proportion of change
SNNPR: South Nations Nationalities and Peoples Region
UC: Umbilical Cord
UCS: Umbilical cord stump
WHO: World Health Organization
